# Identification of key biomarkers associated with development and prognosis in patients with ovarian carcinoma: evidence from bioinformatic analysis

**DOI:** 10.1186/s13048-019-0578-1

**Published:** 2019-11-15

**Authors:** Jiayu Shen, Shuqian Yu, Xiwen Sun, Meichen Yin, Jing Fei, Jianwei Zhou

**Affiliations:** 1 Department of Gynecology, The second affiliated hospital of Zhejiang University School of Medicine, No88, Jiefang Road, Shangcheng District Hangzhou, Zhengjiang, 310002 People’s Republic of China; 2 Department of Gynecology, Tongde hospital of Zhejiang Province, No234, Gucui Road, Xihu District Hangzhou, Zhejiang, 310012 People’s Republic of China; 3 Department of Obstetrics, The Second Affiliated Hospital of Zhejiang University School of Medicine, No88, Jiefang Road, Shangcheng District Hangzhou, Zhengjiang, 310002 People’s Republic of China

**Keywords:** Ovarian cancer, Differentially expressed genes, Bioinformatic analysis

## Abstract

**Background:**

Ovarian cancer (OC) is the deadliest cause in the gynecological malignancies. Most OC patients are diagnosed in advanced stages with less than 40% of women cured. However, the possible mechanism underlying tumorigenesis and candidate biomarkers remain to be further elucidated.

**Results:**

Gene expression profiles of GSE18520, GSE54388, and GSE27651 were available from Gene Expression Omnibus (GEO) database with a total of 91 OC samples and 22 normal ovarian (OV) tissues. Three hundred forty-nine differentially expressed genes (DEGs) were screened between OC tissues and OV tissues via GEO2R and online Venn software, followed by KEGG pathway and gene ontology (GO) enrichment analysis. The enriched functions and pathways of these DEGs contain male gonad development, cellular response to transforming growth factor beta stimulus, positive regulation of transcription from RNA polymerase II promoter, calcium independent cell-cell adhesion via plasma membrane cell adhesion molecules, extracellular matrix organization, pathways in cancer, cell cycle, cell adhesion molecules, PI3K-AKT signaling pathway, and progesterone mediated oocyte maturation. The protein-protein network (PPI) was established and module analysis was carried out using STRING and Cytoscape. Next, with PPI network analyzed by four topological methods in Cytohubba plugin of Cytoscape, 6 overlapping genes (*DTL, DLGAP5, KIF15, NUSAP1, RRM2*, and *TOP2A*) were eventually selected. GEPIA and Oncomine were implemented for validating the gene expression and all the six hub genes were highly expressed in OC specimens compared to normal OV tissues. Furthermore, 5 of 6 genes except for *DTL* were associated with worse prognosis using Kaplan Meier-plotter online tool and 3 of 6 genes were significantly related to clinical stages, including *RRM2, DTL*, and *KIF15*. Additionally, cBioPortal showed that *TOP2A* and *RRM2* were the targets of cancer drugs in patients with OC, indicating the other four genes may also be potential drug targets.

**Conclusion:**

Six hub genes (*DTL, DLGAP5, KIF15, NUSAP1, RRM2*, and *TOP2A*) present promising predictive value for the development and prognosis of OC and may be used as candidate targets for diagnosis and treatment of OC.

## Introduction

Ovarian cancer (OC) is the leading cause of death in gynecological malignancies. There were 22,530 new diagnoses in the United States in 2019. Due to lack of representative symptoms and sensitive diagnostic methods, more than 70% of patients are diagnosed with advanced disease (FIGO III or IV, The International Federation of Gynecology and Obestetrics) as defined by the spread of disease outside the pelvis. The standard treatment remains appropriate surgical staging and debulking surgery, followed by platinum-based systematic chemotherapy. Despite that standard treatment and current novel therapies (such as anti-angiogenesis agents and PARP inhibitors) do improve patients’ outcome and reduce the mortality, the five-year survival is still low (about 47%) due to frequent relapse and drug resistance [1]. Therefore, it is of vital importance and urgency to better understand the mechanism underlying tumorigenesis in OC and develop new strategies for early diagnosis, disease monitoring, and prognosis evaluation.

It is well-known that tumorigenesis is a heterogeneous disease characterized of various gene aberrations, so does ovarian carcinoma. However, the underlying mechanisms of OC development have not been fully understood. Over the past decades, an array of high-throughput technologies for measuring RNA intermediates and epigenetic markers, such as DNA methylation and histone modifications, are widely available. With the continuously rapid development of microarray technology and bioinformatics analysis, genetic alterations at genome level have been widely dug out to identify the differentially expressed genes (DEGs) and functional pathways related to tumorigenesis and prognosis. In the present study, microarray data of three gene expression profiles were downloaded and differentially expressed genes (DEGs) were identified between OC and normal ovarian (OV) tissues, followed by further assessment using Gene Ontology (GO) and Kyoto Encyclopedia of Genes and Genomes (KEGG) pathway. Furthermore, protein-protein interaction (PPI) network of DEGs was established and Cytohubba plugin of Cytoscape was applied for identifying some core genes. Moreover, the expression of the overlapping genes between OC and normal OV tissues were validated using Gene Expression Profiling Interactive Analysis (GEPIA) and Oncomine. Taken together, six DEGs were selected for further analysis, namely *DTL, DLGAP5, KIF15, NUSAP1, RRM2,* and *TOP2A*. Then, the Kaplan Meier plotter online tool was used to assess the prognostic value of these core genes, showing that 5 genes, except for *DTL*, were correlated with worse survival. Three of those six hub genes were found to be significantly differentiated in various clinical stages, including *RRM2, DTL*, and *KIF15*. In addition, to explore relationships between genes and drugs, CBio Cancer Genomics Portal (cBioPortal) was used and showed that *TOP2A* and *RRM2* were the targets of cancer drugs in patients with OC, indicating the other four genes may also be potential drug targets. In conclusion, this bioinformatic study provides some promising biomarkers associated with development and prognosis in patients of OC.

## Materials and methods

### Microarray data

GEO (http://www.ncbi.nlm.nih.gov/geo) functions as a public functional genomics database of high throughout gene expression data, chips and microarrays [2]. Three gene expression profiles in OC and normal OV tissues were downloaded from GEO, that is GSE18520 [3], GSE54388 [4] and GSE27651 [5]. Microarray data of these three datasets were all on account of GPL570 Platforms, [HG-U133_Plus_2] Affymetrix Human Genome U133 Plus 2.0 Array. The GSE18520 dataset contained 53 high grade OC samples and 10 normal OV samples. GSE54388 contained 16 high grade OC samples and 6 normal OV samples while GSE27651 contained 22 high grade OC specimen and 6 normal OV specimen.

### Identification of DEGs

GEO2R (http://www.ncbi.nlm.nih.gov/geo/geo2r) is regarded as an interactive online tool designed to compare two or more datasets in a GEO series for the purpose of DEGs identification across experimental conditions. The DEGs between OC tissues and normal OV tissues were identified using GEO2R with the threshold of |logFC| > 2 and *P* value < 0.05 which were considered of statistically significance. For the next step, the online Venn software was applied to detect the intersection DEGs among three datasets.

### Functional enrichment analysis

The GO datasets and KEGG pathway enrichment were used to analyze DEGs at the functional level with DAVID (The Database for Annotation, Visualization and Integrated Discovery, http://david.ncifcrf.gov/,version 6.8) [6–8]. DAVID is a comprehensive database of functional annotation tools for connecting functional terms with gene lists using a clustering algorithm. In order to elucidate the functional profiles of the DEGs, we used DAVID to obtain the enriched biological process (BP), cellular component (CC), molecular function (MF) and KEGG pathway. *P* < 0.05 was considered statistically significant.

### PPI network construction and module analysis

STRING (Search Tool for the Retrieval of Interacting Genes, http://string-db.org, version 11.0) online database was used to predict the PPI network which may further explain the mechanisms of the occurrence and progression of diseases [9]. By using STRING database, PPI network of DEGs was analyzed and an interaction with a combined score > 0.4 was recognized as statistical significance. The plug-in MCODE (Molecular Complex Detection) app of Cytoscape (an public bioinformatics software, version 3.7.1) is constructed for clustering a network based on topology to determine intensively connected regions [10, 11]. The PPI network was plotted with the application of Cytoscape and the most significant module in the PPI network was narrowed down using MCODE with the following criteria: degree cutoff =10, node score cutoff = 0.2, k-core = 2, max depth = 100.

### Hub genes selection and analysis

The plug-in Cytohubba of Cytoscape is an APP provided with 11 topological analysis methods for ranking nodes in a PPI network by their network features [12]. In the present study, the top 20 hub genes were ranked according to the maximal clique centrality (MCC), maximum neighborhood component (MNC), Degree and edge percolated component (EPC). The overlapping hub genes in top 20 by these four topological methods were selected for further bioinformatics analysis using the GeneMANIA App of Cytoscape which contains a comprehensive sets of datasets from GEO, BioGRID, Pathway Commons and I2D, as well as organism specific functional genomics datasets [13]. Meanwhile, the biological process of hub genes was also visualized using BiNGO (Biological Networks Gene Oncology tool, version 3.0.3) plugin of Cytoscape [14]. Furthermore, GEPIA (http://gepia.cancer-pku.cn/index.html) website and online database Oncomine (http://www.oncomine.com) were both applied for validating the gene expression [15, 16]. GEPIA is a web-based tool to provide key interactive and customizable functions based on TCGA (The Cancer Genome Atlas) and GTEx (Genotype-Tissue Expression) data. The overall survival of hub genes was analyzed using Kaplan Meier-plotter online tool which is commonly applied for assessing the effect of genes on survival based on EGA, TCGA database and GEO [17]. CBioPortal was used for exploring genetic alterations of hub genes and relationships between genes and drugs [18].

## Result

### Identification of DEGs in ovarian cancer

**Fig. 1:**
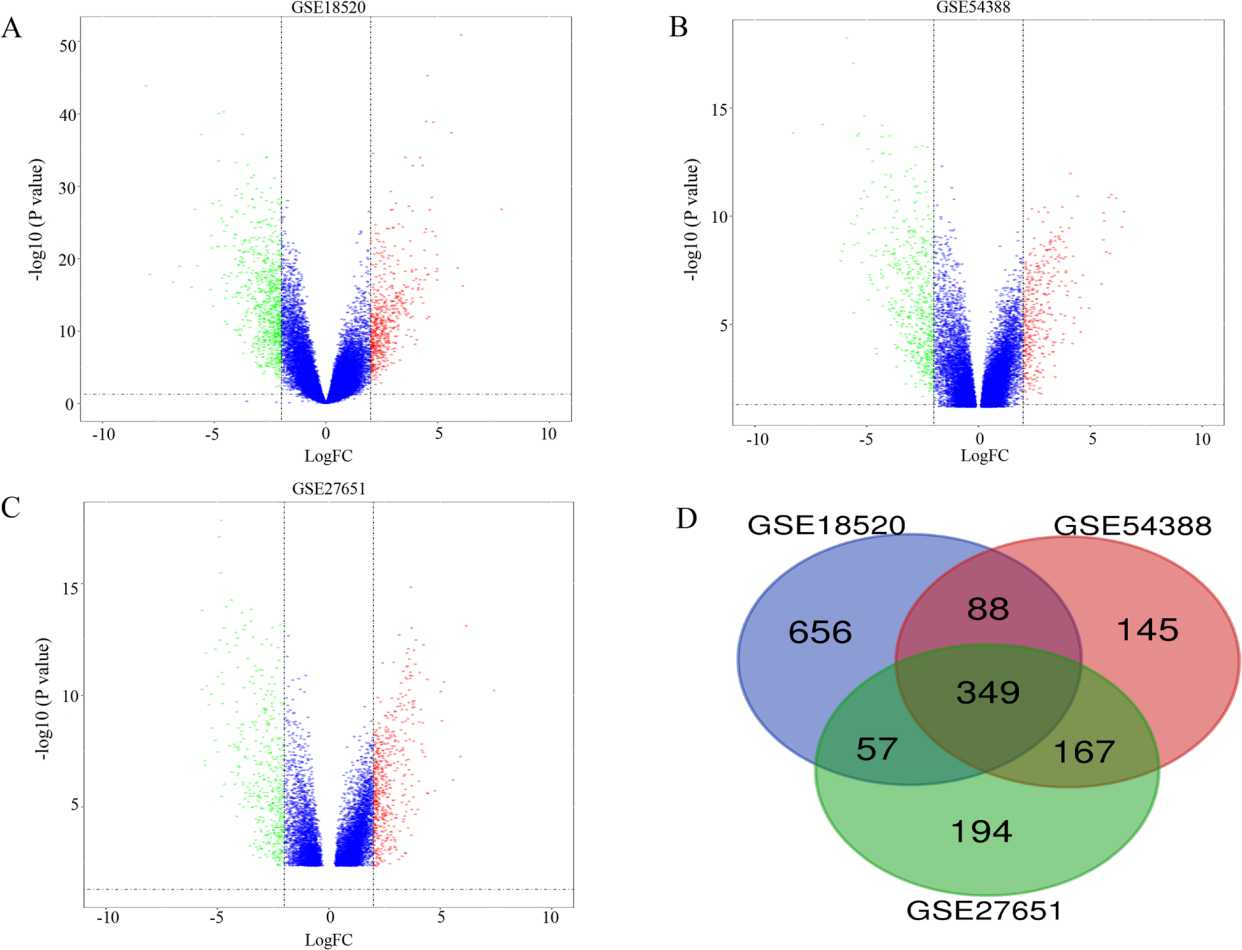
**a**-**c** Volcano plot of DEGs between OC tissues and normal OV tissues in each dataset. Red dots: significantly up-regulated genes in OC; Green dots: significantly down-regulated genes in OC; Blue dots: non-differentially expressed genes. *P* < 0.05 and |logFC| > 2 were considered as statistically significant. **d** Venn diagram of overlapping 349 DEGs from GSE18520, GSE54388 and GSE27651 datasets

Via GEO2R online tools, DEGs in three datasets (1273 DEGs in GSE18520, 910 in GSE54388, and 905 in GSE27651, respectively) were extracted after gene expression profile data processing and standardization with the cutoff standard of *P* value < 0.05 and |logFC| > 2 (Fig. 1a-c). The overlapping DEGs among these three datasets contained 349 genes as shown in the Venn diagram (Fig. 1d).

### Enrichment analysis for DEGs

**Fig. 2:**
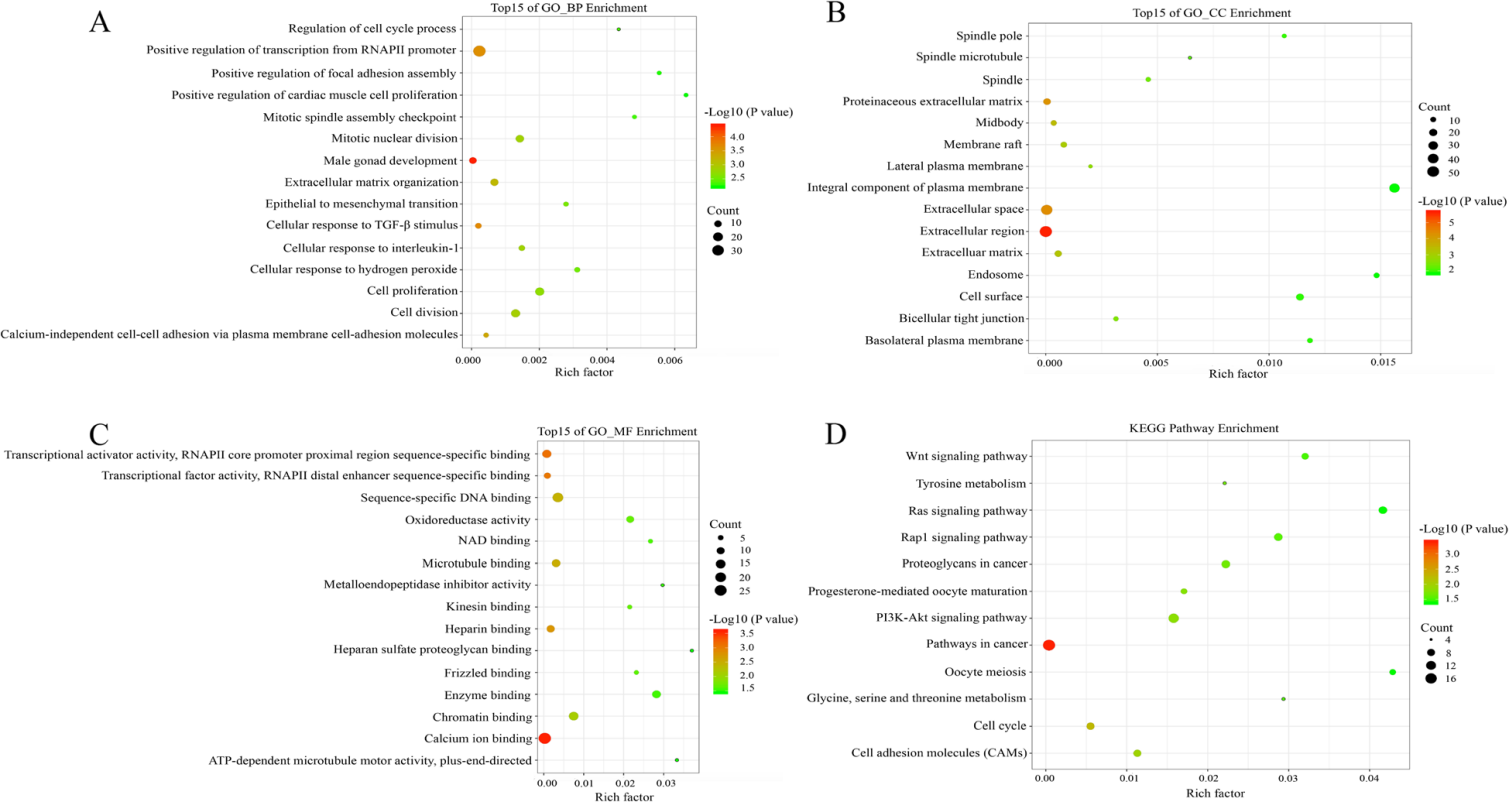
GO and KEGG analysis of the overlapping DEGs in OC. **a** Biological process. **b** Cellular component. **c** Molecular function. **d** KEGG pathway. All of the enrichment pathways were generated using the ggplot2 package in R language

To elucidate the biological functions of the overlapping DEGs, we performed functional annotation and pathway enrichment analysis via DAVID online tool. Results indicated that the overlapping DEGs in biological process of GO enrichment were markedly associated with male gonad development, cellular response to transforming growth factor beta (TGFβ) stimulus, positive regulation of transcription from RNA polymerase II (RNAP II) promoter, calcium independent cell-cell adhesion via plasma membrane cell adhesion molecules, and extracellular matrix organization (Fig. 2a). As for molecular function of GO enrichment, DEGs were remarkably related to calcium ion binding, transcriptional activator activity (RNAP II core promoter proximal region sequence specific binding), transcriptional factor activity (RNAP II distal enhancer sequence specific binding), heparin binding, and microtubule binding (Fig. 2b). In addition to cellular component, the overlapping DEGs were particularly enriched in extracellular region, extracellular space, proteinaceous extracellular matrix, midbody, and extracellular matrix (Fig. 2c). Besides, signaling pathway analysis of KEGG demonstrated that those DEGs played pivotal roles in pathways in cancer, cell cycle, cell adhesion molecules, PI3K-AKT signaling pathway, and progesterone mediated oocyte maturation (Fig. 2d).

### PPI network construction and significant module identification

**Fig. 3:**
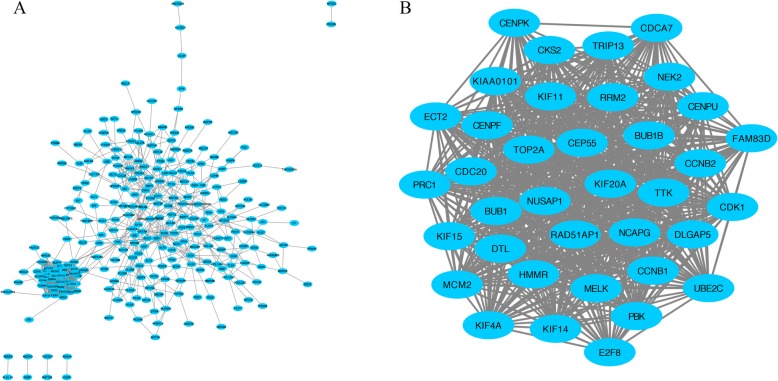
Common DEGs PPI network construction and module analysis. **a** A total of 270 DEGs were visualized in the DEGs PPI network complex: the nodes represent proteins, the edges represent the interaction of the proteins. **b** Module analysis using MCODE: degree cutoff =10, node score cutoff = 0.2, k-core = 2, max depth = 100


STRING database was used to predict the potential relationships among these overlapping DEGs at protein levels with combined score > 0.4. The establishment of the PPI network was constructed via Cytoscape software, including 270 nodes and 1169 edges (Fig. 3a). Additionally, the most important PPI network modules were obtained using MCODE, consisted of 37 nodes and 640 edges (Fig. 3b). To further identify the hub genes, we applied Cytohubba plugin of Cytoscape for ranking the top 20 nodes in the above PPI network according to four topological analysis methods, including MCC, MNC, Degree, and EPC (Table 1). A total of 6 overlapping hub genes were determined for further analysis, namely *DTL, DLGAP5, KIF15, NUSAP1, RRM2,* and *TOP2A*.

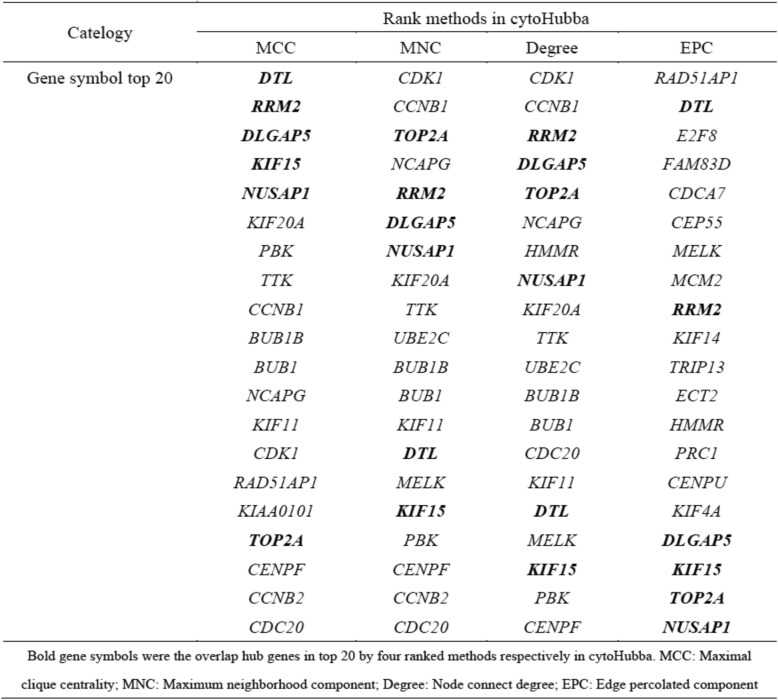

**Table 1:** Hub genes for highly differentiated expressed genes ranked in Cytohubba plugin of Cytoscape

Catelogy	Rank methods in cytoHubba			
	MCC	MNC	Degree	EPC
Gene symbol top 20	***DTL***	*CDK1*	*CDK1*	*RAD51AP1*
	***RRM2***	*CCNB1*	*CCNB1*	***DTL***
	***DLGAP5***	***TOP2A***	***RRM2***	*E2F8*
	***KIF15***	*NCAPG*	***DLGAP5***	*FAM83D*
	***NUSAP1***	***RRM2***	***TOP2A***	*CDCA7*
	*KIF20A*	***DLGAP5***	*NCAPG*	*CEP55*
	*PBK*	***NUSAP1***	*HMMR*	*MELK*
	*TTK*	*KIF20A*	***NUSAP1***	*MCM2*
	*CCNB1*	*TTK*	*KIF20A*	***RRM2***
	*BUB1B*	*UBE2C*	*TTK*	*KIF14*
	*BUB1*	*BUB1B*	*UBE2C*	*TRIP13*
	*NCAPG*	*BUB1*	*BUB1B*	*ECT2*
	*KIF11*	*KIF11*	*BUB1*	*HMMR*
	*CDK1*	***DTL***	*CDC20*	*PRC1*
	*RAD51AP1*	*MELK*	*KIF11*	*CENPU*
	*KIAA0101*	***KIF15***	***DTL***	*KIF4A*
	***TOP2A***	*PBK*	*MELK*	***DLGAP5***
	*CENPF*	*CENPF*	***KIF15***	***KIF15***
	*CCNB2*	*CCNB2*	*PBK*	***TOP2A***
	*CDC20*	*CDC20*	*CENPF*	***NUSAP1***

Bold gene symbols were the overlap hub genes in top 20 by four ranked methods respectively in cytoHubba. *MCC* Maximal clique centrality, *MNC* Maximum neighborhood component, *Degree* Node connect degree, *EPC* Edge percolated component

### Re-analysis of the six selected genes

**Fig. 4:**
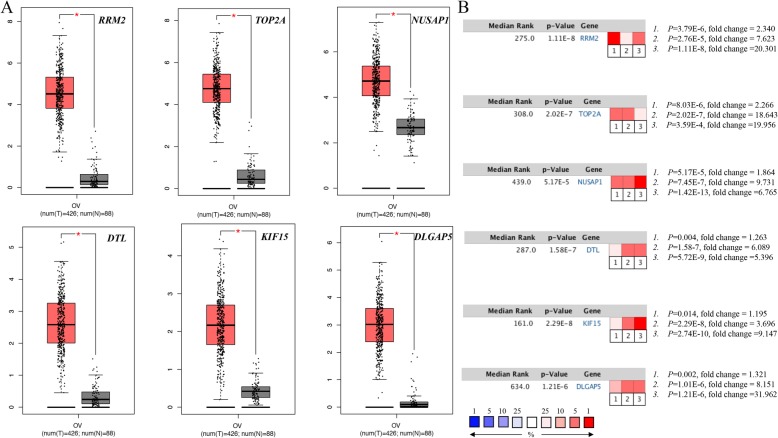
**a** The expression level of hub genes between OC and normal OV tissue according to GEPIA database. **b** Oncomine analysis of cancer vs. normal tissue of hub genes. Heat maps of hub gene expression in clinical OC specimen vs. normal OV tissues: 1.Ovarian serous Adenocarcinoma vs. Normal, Lu Ovarian, Clin Cancer Res, 2004; 2.Ovarian serous cystadenocarcinoma vs. Normal, TCGA Ovarian, 2013; 3.Ovarian serous adenocarcinoma vs. Normal, Yoshihara Ovarian, Cancer Sci, 2009

**Fig. 5:**
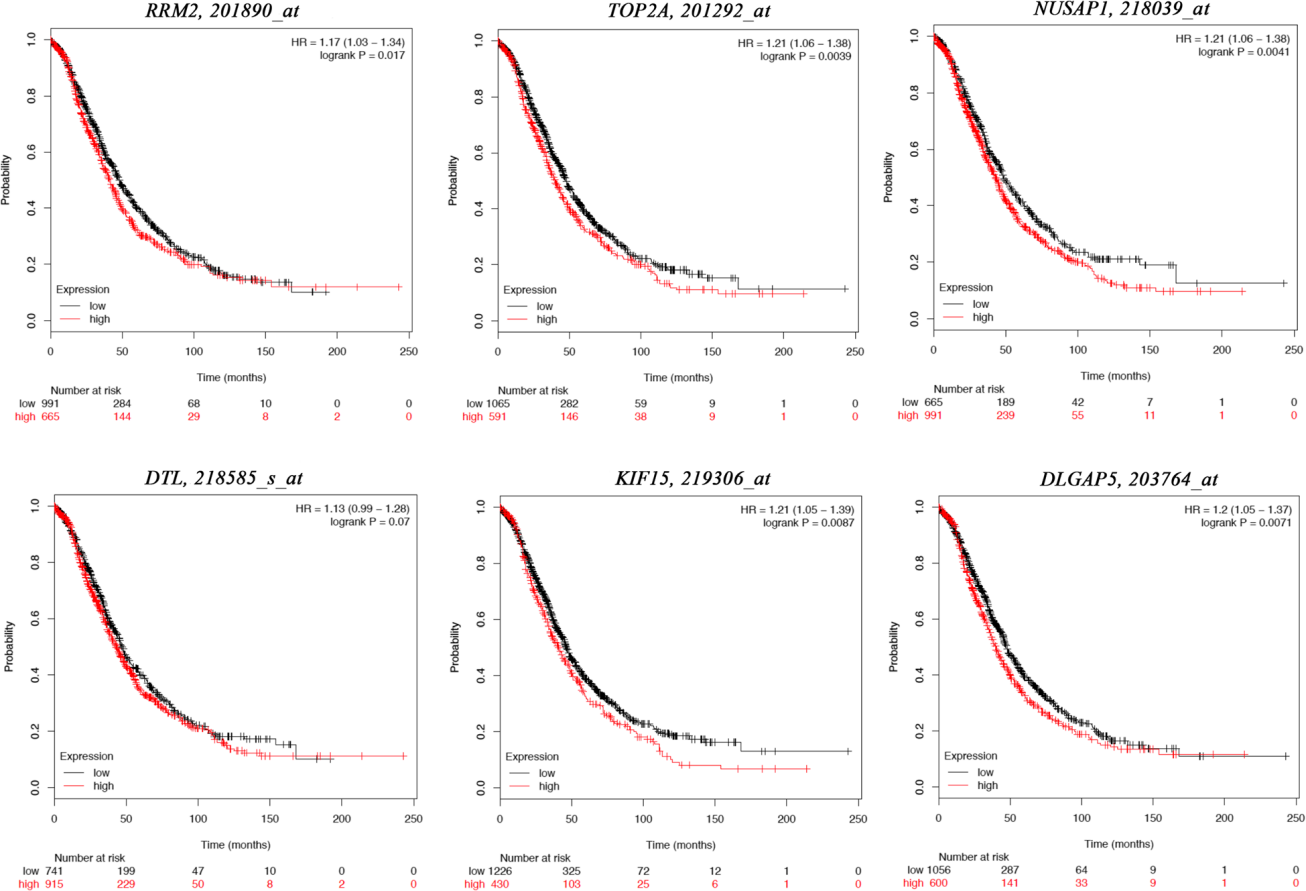
The prognostic information of the 6 hub genes. The online tool Kaplan Meier plotter was applied for identification of the prognostic value of hub genes and 5 of 6 were correlated with worse survival. (*P* < 0.05)

**Fig. 6:**
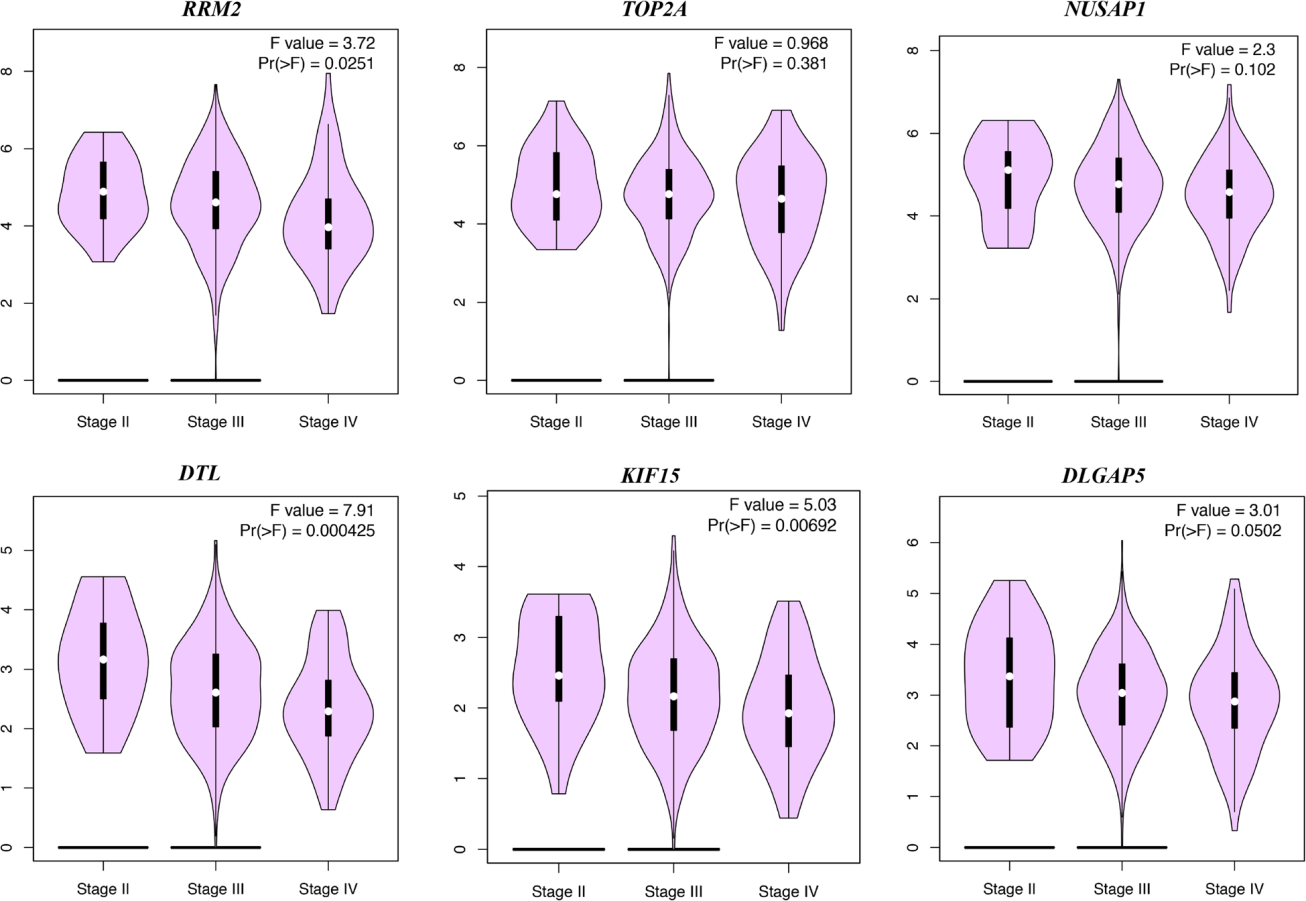
Validation of the differential expression of six hub genes in various clinical stages. ANOVA was used to assess the statistical significance of the differences. Results showed that 3 of 6 hub genes were significantly differentiated in various clinical stages

**Fig. 7:**
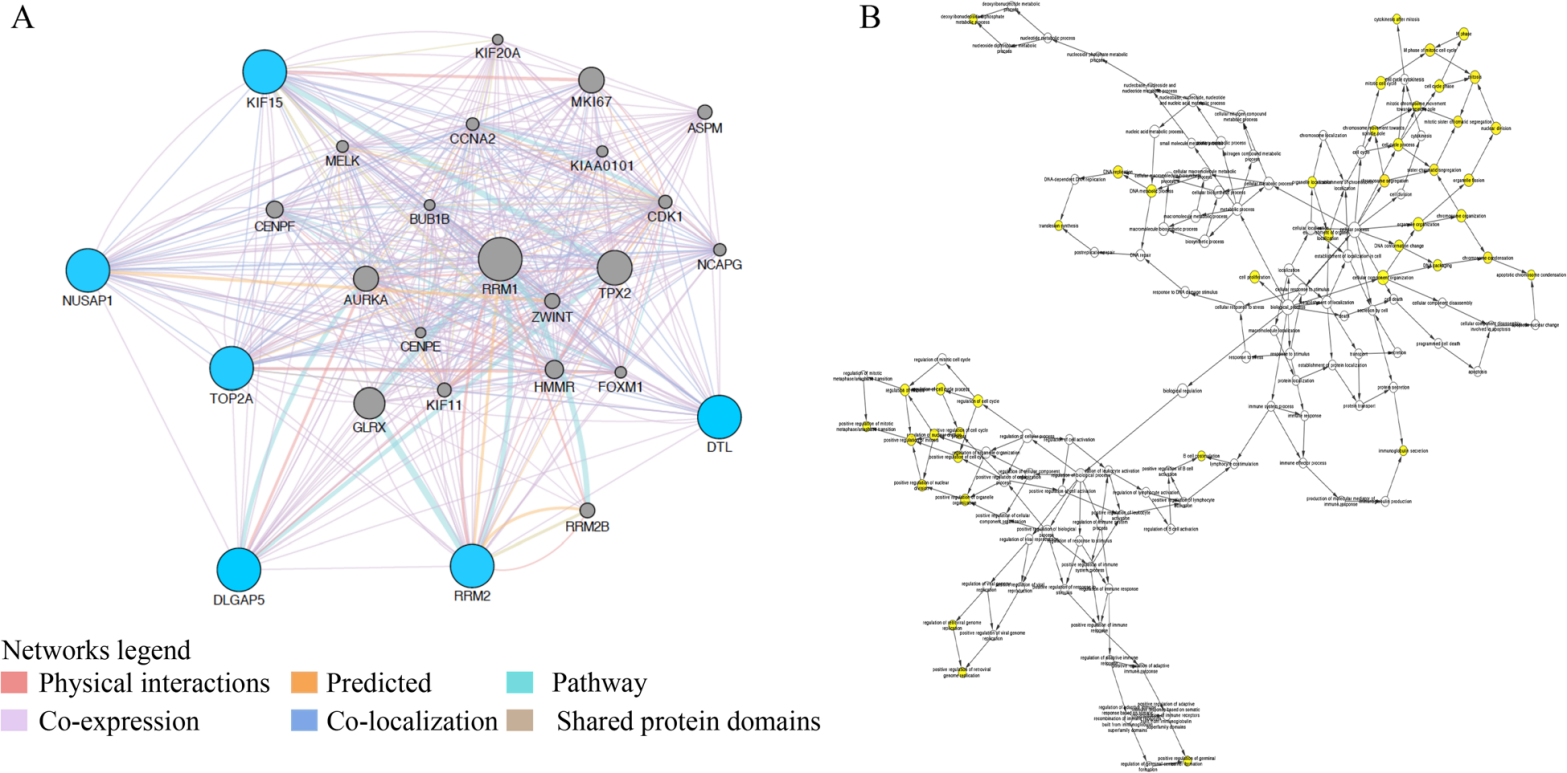
**a** The network of hub genes and their related genes constructed by GeneMANIA. **b** The biological process of hub genes analyze by BiNGO (*P* < 0.01). The color depth of node represents the corrected *P*-value. The size of nodes represents the number of genes involved

**Fig. 8:**
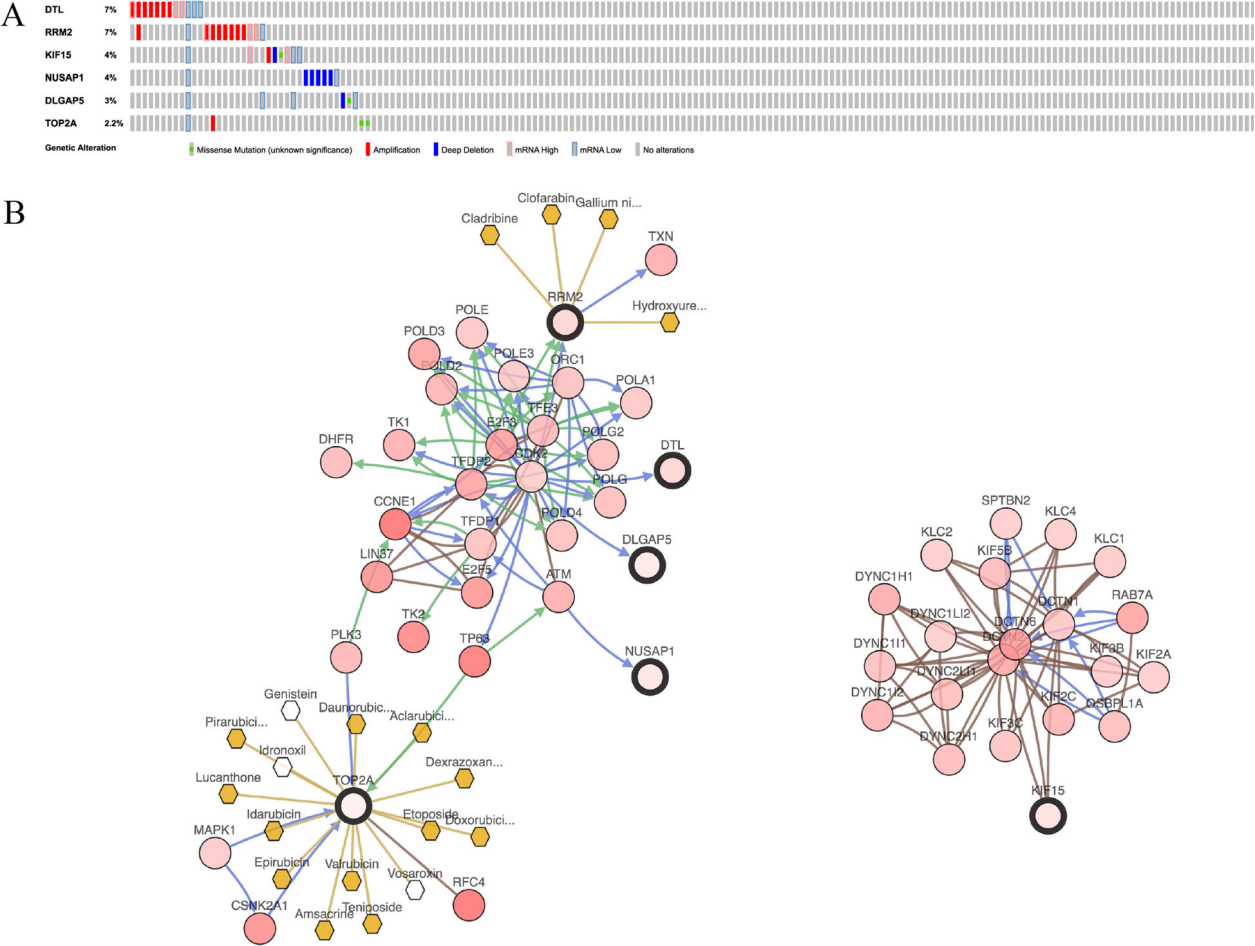
Genetic alterations associated with hub genes in TCGA-OC. **a** A visual summary across on a query of six hub gene showing genetic alteration of six hub genes in TCGA-OC patients. **b** The network contains 56 genes (6 hub genes and 50 most variant genes). Relationship between hub genes and tumor drugs is also illustrated

GEPIA and Oncomine tool were used to further validate the expression of these 6 genes. Both the databases verified that the expression of *DTL, DLGAP5, KIF15, NUSAP1, RRM2,* and *TOP2A* presented significant dissimilarities in OC samples and normal OV samples (Fig. 4). Via Kaplan Meier plotter online tool, we found that five of the six selected genes, except for *DTL*, were correlated with worse survival (Fig. 5). The training dataset (TCGA-OV) was applied to validate the correlations between the six hub genes and clinical stages. Three of those six hub genes were found to be negatively related to clinical stages, including *RRM2, DTL*, and *KIF15* (Fig. 6). A protein/gene interaction network for the six genes and their products with 20 proteins/ genes was generated via GeneMANIA plugin of Cytoscape, including *RRM1, TPX2, GLRX, MKI67, AURKA, HMMR, CENPF, ZWINT, RRM2B, ASPM, KIF11, CDK1, CCNA2, NCAPG, MELK, FOXM1, KIAA0101, BUB1B, KIF20A,* and *CENPE* (Fig. 7a)*.* The ranking order based on the score ranging from high to low was *NUSAP1, DTL, KIF15, DLGAP, RRM2,* and *TOP2A*. Besides, the biological process analysis of the hub genes was visualized in Fig. 7b, indicating the top 5 involved BPs includes chromosome segregation, positive regulation of nuclear division, positive regulation of mitosis, DNA replication, and chromosome condensation. For genetic alteration, six hub genes were altered in 39 (21%) of 182 patients. Among the six genes, *DTL* and *RRM2* altered most (both were 7%) and the main type was amplification (Fig. 8a). Besides, regarding with the relationship between anticancer drugs and hub genes, *TOP2A* and *RRM2* were the targets of cancer drugs in patients with OC (Fig. 8b).

## Discussion

Ovarian cancer remains the deadliest cause among malignancies of the female reproductive system. Long-term survival of OC patients is still unsatisfactory as a result of late diagnosis, recurrence and drug resistance. Early diagnosis plays a crucial role in the prevention and prognosis of cancers, including ovarian carcinoma. Cancer antigen 125 (CA125) have been most widely used in diagnosis and monitoring in OC patients [19, 20]. Anyhow, not all the OC patients present with abnormal CA125 level. Though human epididymis protein 4 (HE4) is also approved by the US Food and Drug Administration (FDA) for monitoring, HE4 is not applied in routine practice due to contradictory studies [21]. Others, like circulating cell-free DNA (cfDNA), have also been regarded as diagnostic and prognostic markers for ovarian cancer [22]. However, isolation and detection techniques of these biomarkers limit their use in most cases. Thus, it is urgent to find new effective biomarkers.

In the present study, the microarray datasets of GSE18520, GSE54388, and GSE27651 were chosen to identify DEGs between OC and normal OV tissues with a total of ninety-one OC samples and twenty-two normal samples enrolled. The integrated results revealed 349 commonly changed genes that were significantly abnormally expressed in ovarian cancer specimens (*P* < 0.05, |logFC| > 2). Then, GO and KEGG enrichment analysis were carried out to dig up the biological functions and signaling pathways of these DEGs. For BP, DEGs were significantly associated with male gonad development, cellular response to TGFβ stimulus, positive regulation of transcription from RNAP II promoter, calcium independent cell-cell adhesion via plasma membrane cell adhesion molecules, and extracellular matrix organization. Among the MF, DEGs were primarily related to calcium ion binding, transcriptional activator activity, transcriptional factor activity, heparin binding, and microtubule binding. In terms of cellular component, these DEGs were particularly enriched in extracellular region, extracellular space, proteinaceous extracellular matrix, midbody, and extracellular matrix. Furthermore, KEGG pathway enrichment revealed remarkable involvement of DEGs in pathways in cancer, cell cycle, cell adhesion molecules, PI3K-AKT signaling pathway, and progesterone mediated oocyte maturation. Increasing evidence implies that PI3K-AKT pathway participates in OC proliferation, migration process and chemoresistance [23–25]. Hyer-activated PI3K-AKT pathway plays a central role in cancer cell metabolic adaptation since its downstream effectors control most of the glycolytic and glutaminolysis genes. Previous studies indicated that PI3K regulated G1 cell cycle and apoptosis in ovarian cancer via stimulating AKT/mTOR/p70S6K1 signaling [24]. Except for PI3K-AKT, dysregulation of TGFβ pathway has also been well studied and implicated in various tumorigenesis and progression, including ovarian malignancy. Studies showed that TGFβ could create an environment where ovarian cancer cell can evade the host immune defense resulting in tumor dissemination and worse outcomes in patients with OC [25]. Generally speaking, all above theories were in accordance with our bioinformatics analysis results. After further analysis of DEGs PPI network, six hub genes including *DTL, DLGAP5, KIF15, NUSAP1, RRM2,* and *TOP2A* were filtered out which were all significantly unregulated in OC tissue compared to normal OV tissues. In addition, GEPIA and Oncomine were applied for further validation of the expression levels of these key genes in OC. Undoubtedly, both databases demonstrated the same trend on expression as presented by bioinformatics analysis. Using the data from Kaplan Meier plotter, we noted that OC patients with high expression of *DLGAP5, KIF15, NUSAP1, RRM2,* and *TOP2A* had worse survival outcomes while *DTL* made no statistical difference. Three of those six hub genes, including *RRM2, DTL*, and *KIF15,* were negatively associated with clinical stages while the other three genes showed no statistical difference. Though TCGA-OV datasets showed the expression trends of *RRM2, DTL*, and *KIF15* were downregulated in advanced stages, the overall expression levels of these three genes in OC were upregulated when compared to normal tissues, suggesting other regulatory mechanisms might be involved which required further studies. Finally, we fabricated a protein/gene interaction network for the six genes and their products with 20 proteins/ genes via GeneMANIA and re-analyzed the biological process via BiNGO, suggesting that the hub genes played pivotal roles in chromosome segregation, positive regulation of nuclear division, positive regulation of mitosis, DNA replication, and chromosome condensation. These result indicated that these genes and pathways may exert important roles in the occurrence and progression of OC.

DTL (denticleless E3 ubiquitin protein ligase), also known as CDT2/RAMP/DCAF2/L2DTL, has an oncogenic function in several cancer types, such as breast cancer, hepatocellular carcinoma (HCC), gastric cancer, and Ewing sarcoma [26–30]. It is also been reported that DTL has a profound effect on regulating the protein stability of P53 which is regarded as a tumor suppressor and regulates cell cycle progression and cell survival. Banks et al. reported that inactivation of DTL contributes to P53 stabilization and cell growth arrest. In unstressed Hela cells, p53 stabilization was induced by knockdown of DTL/RAMP leading to the accumulation of G2/M cells [29]. Similarly, knockout of DTL has been demonstrated to inhibit cell proliferation, migration, and invasion of gastric cancer cells in a TP53 mutation independent manner, as the study conducted by Kobayashi et al. showed [30]. These results suggested that DTL could be treated as a valuable biomarker and target for a wide range for cancers, including OC.

DLGAP5, disc large homolog associated protein 5, has a pivotal role in spindle assembly and chromosomal segregation during mitosis, which has been found to be unregulated in several cancer types, including OC, colorectal cancer (CRC), HCC, and adrenocortical tumors [31–34]. Depletion of DLGAP5 can lead to prolonged prometaphase and aberrant chromatin segregation. Branchi et al. found that downregulation of DLGAP5 remarkably inhibited the invasion and migration ability of colorectal cancer cells and overexpression of DLGAP5 was associated with poor overall survival in CRC patients [32]. Overexpression of DLGAP5 induces the accumulation of the oncoprotein Gankyrin leading to the ubiquitination and degradation of P53 [33]. In prostate cancer, studies indicated that loss of DLGAP5 gene sensitizes androgen-dependent LNCaP cells to docetaxel treatment due to lower density of microtubule in their central spindles which requires a lower molecule content of drug to bind and stabilize the microtubule, suggesting that DLGAP5 may provide a potential novel target for chemotherapy efficacy [34]. In OC, considerable evidence shows an important oncogenic role of Notch signaling [35–37]. Recent large-scale genomic and epigenetic analysis of TCGA revealed that altered Notch signaling in 22% of cases diagnosed of OC with alterations in Notch3 occurred in 50% of those cases [36]. Chen et al. found that DLGAP5 was a direct target genes of Notch3 in OC. Ectopic expression of DLGAP5 can partially reverse the antiproliferative effect of Notch3 pathway inactivation. In contrast, DLGAP5 knockdown in OC cells inhibited cellular proliferation and tumorigenesis by arresting the cell cycle at the G2-M phase [37]. Taken together, DLGAP5 may present promising predictive value for the development and prognosis of OC.

KIF15 (kinesin family member15), which belongs to the kinesin 12 family, plays an important role in promoting cell mitotic and cellular material transportation [38]. In melanoma, KIF15 was found to be significantly upregulated in cancer cells and tissues and the suppression of KIF15 remarkably reduced tumor growth and increased the apoptosis of cancer cells [39]. Knockdown of KIF15 in Hela cells made it incapable of developing resistance to Eg5 inhibitors, suggesting KIF15 may be indispensable for Eg5 inhibitors resistance [40]. Additionally, study found that 26 of 38 kinesins detected in breast cancer MCF-7 cells are regulated by estrogen 17β-estradiol (E2) and many of them are upregulated by E2, including KIF15, KIF4A, KIF20A, and KIF23. Further study showed that multiple kinesins including above four kinesins plays important roles in the growth and survival of both tamoxifen-sensitive and resistant breast cancer cells and high levels of the four kinesins are strongly related to poor recurrence-free survival in patients treated with tamoxifen, suggesting that kinesins like KIF15 present crucial values in predicting prognosis and may be used as therapeutic targets for breast cancer [41]. Considering part of breast cancer women have genetic predisposition to OC, we assumed that KIF15 may also be responsible for tumorigenesis and can be regarded as therapeutic targets in OC.NUSAP1, nucleolar and spindle-associated protein 1, is a kind of microtubule and chromatin binding protein involved in multiple cancer cell proliferation, migration, and invasion. The transcript level of NUSAP1 was positively related to E2F1 but negatively related to RB1. Moreover, it was found that NUSAP1 promotes the invasion, migration, and metastasis of prostate cancer cells by regulating FAM101B which is regarded as a TGFβ1 signaling effector related to epithelial to mesenchymal transition (EMT) [42]. Studies also showed that the over-expression of NUSAP1 stimulated sumoylation of TCF4 via interacting with SUMO E3 ligase Ran-binding protein 2 and hyperactivated Wnt/β-caternin signaling to induce cancer stem cell properties and EMT, and finally promoted the metastasis of cervical cancer [43]. Zhang et al. silenced the NUSAP1 gene in MCF-7 cells and results showed that downregulation of NUSAP1 reduced the expression of CKD1 and DLGAP5 to inhibit the proliferation and invasion of MCF-7 cells and enhance the drug sensitivity to Epirubicin [44]. In triple-negative breast cancer, the expression level of NUSAP1 was found to be significantly correlated with *BRCA1* expression and higher expression of NUSAP1 led to poor prognosis while positive *BRCA1* was related to improved outcomes [45, 46]. Considering *BRCA1* is well-known to be associated with ovarian cancer, we assumed that NUSAP1 could also be a promising biomarker for OC.

RRM2, ribonucleotide reductase regulatory subunit M2, functions actively in promoting cell invasion, migration, and tumor metastasis. Wang et al. found that over-expression of RRM2 mediated by HPVE7 facilitated tumor growth and angiogenesis in cervical cancer, consistent with the experimental results carried out by Zhao et al. which showed that silencing of RRM2 enhanced the apoptosis and suppressed the tumorigenic ability of Hela cells [47, 48]. Additionally, studies conducted by Rahman et al. indicated that over-expression of RRM2 was associated with induced resistance to chemotherapeutic agent cisplatin [49]. Taken together, we assumed that RRM2 may be a potent biomarker and target for the tumorigenesis and drug therapy.

TOP2A, topoisomerase (DNA) II alpha, accumulates on chromatin during mitosis and targets that mitotic centromere during prophase. TOP2A is found to exert functions in DNA stability and act as one of the targets of chemotherapeutic agents including anthracyclines and etoposide [50–52]. Ghisoni et al., reported that in patients with platinum-resistant/partially platinum-sensitive epithelial ovarian cancer, TOP2A expression over 18% was correlated with a higher sensitivity to pegylated liposomal doxorubicin (PLD). Besides, patients with TOP2A expression above the cut-off who treated with PLD monotherapy reached a longer time to progression compared with PLD-doublet therapy [53]. All above data suggested the promising value of TOP2A in predicting activity of PLD in OC patients.

Previous studies showed that small molecules might have a beneficial effect on disease, making it possible to present genes as therapeutic targets [54]. One study showed that mesothelin, FLT4, α-1 acid glycoprotein (AGP) and CA125 may be potential markers for patients with OC who are more likely to benefit from bevacizumab [55]. Several studies indicated that OC patients with germline or somatic *BRCA1* or *BRCA2* mutations show benefit from PARP inhibitors [56–58]. In order to explore new targets for anticancer drugs in OC patients, we applied cBioPortal to elucidate the relationship between these six hub genes and cancer drugs. Results showed that *TOP2A* and *RRM2* were previously demonstrated to be the targets for anticancer drugs, suggesting the remaining genes (*DTL, DLGAP5, KIF15*, and *NUSAP1*) might also have promising value in serving as drug targets.

Although meaningful insights were found in this study, there are some limitations. First of all, lack of experimental validation might be the biggest limitation of our study. Secondly, the mechanism of how these six genes influence the tumorigenesis and progression of OC remains unclear. Therefore, further investigations are needed to clarify the function and the possible mechanism of these hub genes.

## Conclusion

In conclusion, with the integrated bioinformatics analysis for gene expression profiles in ovarian malignancy, we dug out six core molecules associated with the pathogenesis and prognosis of OC, including *DTL, DLGAP5, KIF15, NUSAP1, RRM2*, and *TOP2A*. These hub genes were all unregulated in OC and four of them may be associated with targeted therapy. These hub genes may be regarded as novel diagnostic and prognostic biomarkers for OC. However, further in-depth study (in vivo and in vitro experiment) is necessary to elucidate the biological function of these genes in ovarian carcinoma.

## Data Availability

The datasets used and analysed during the current study are available from the corresponding author on reasonable request.

## Abbreviations

AGP: α-1 acid glycoprotein
BiNGO: Biological Networks Gene Oncology
BP: Biological process
CA125: Cancer antigen 125
cBioPortal: CBio Cancer Genomics Portal
CC: Cellular component
cfDNA: Circulating cell-free DNA
CRC: Colorectal cancer
DAVID: The Database for Annotation, Visualization and Integrated Discovery
DEGs: Differentially expressed genes
DLGAP5: Disc large homolog associated protein 5
DTL: Denticleless E3 ubiquitin protein ligase
E2: Estrogen 17β-estradiol
EMT: Epithelial to mesenchymal transition
EPC: Edge percolated component
FDA: Food and Drug Administration
FIGO: The International Federation of Gynecology and Obestetrics
GEO: Gene expression omnibus
GEPIA: Gene Expression Profiling Interactive Analysis.
GO: Gene ontology
GTEx: Genotype-Tissue Expression
HCC: Hepatocellular carcinoma
HE4: Human epididymis protein 4
KEGG: Kyoto Encyclopedia of Genes and Genomes
KIF15: Kinesin family member15
MCC: Maximal clique centrality
MCODE: Molecular Complex Detection
MF: Molecular function
MNC: Maximum neighborhood component
NUSAP1: Nucleolar spindle-associated protein 1
OC: Ovarian cancer
OV: Ovarian
PLD: Pegylated liposomal doxorubicin
PPI: Protein-protein interaction
RNAP II RNA: Polymerase II
RRM2: Ribonucleotide reductase regulatory subunit M2
STRING: Search Tool for the Retrieval of Interacting Genes
TCGA: The Cancer Genome Atlas
TGFβ: Transforming growth factor beta
TOP2A: Topoisomerase (DNA) II alpha
